# Survival and long-term outcomes following in-hospital cardiac arrest in a Swiss university hospital: a prospective observational study

**DOI:** 10.1186/s13049-021-00931-0

**Published:** 2021-08-11

**Authors:** Alexander Fuchs, Dominic Käser, Lorenz Theiler, Robert Greif, Jürgen Knapp, Joana Berger-Estilita

**Affiliations:** 1 Department of Anaesthesiology and Pain Medicine, Inselspital, Bern University Hospital, University of Bern, Freiburgstrasse 18, 3010 Bern, Switzerland; 2 Department of Anaesthesia, Kantonsspital Aarau, Aarau, Switzerland; 3 School of Medicine, Sigmund Freud University Vienna, Vienna, Austria; 4 ERC Research NET, Niel, Belgium; 5 Center for Health Technology and Services Research (CINTESIS), Faculty of Medicine of Porto, Porto, Portugal

**Keywords:** In-hospital cardiac arrest, Cardiopulmonary resuscitation, Return of spontaneous circulation, Chain of survival, Functional outcome, Health-related quality of life

## Abstract

**Background:**

Incidence of in-hospital cardiac arrest is reported to be 0.8 to 4.6 per 1,000 patient admissions. Patient survival to hospital discharge with favourable functional and neurological status is around 21–30%. The Bern University Hospital is a tertiary medical centre in Switzerland with a cardiac arrest team that is available 24 h per day, 7 days per week. Due to lack of central documentation of cardiac arrest team interventions, the incidence, outcomes and survival rates of cardiac arrests in the hospital are unknown. Our aim was to record all cardiac arrest team interventions over 1 year, and to analyse the outcome and survival rates of adult patients after in-hospital cardiac arrests.

**Methods:**

We conducted a prospective single-centre observational study that recorded all adult in-hospital cardiac arrest team interventions over 1 year, using an Utstein-style case report form. The primary outcome was 30-day survival after in-hospital cardiac arrest. Secondary outcomes were return of spontaneous circulation, neurological status (after return of spontaneous circulation, after 24 h, after 30 days, after 1 and 5 years), according to the Glasgow Outcomes Scale, and functional status at 30 days and 1 year, according to the Short-form-12 Health Survey.

**Results:**

The cardiac arrest team had 146 interventions over the study year, which included 60 non-life-threatening alarms (41.1%). The remaining 86 (58.9%) acute life-threatening situations included 68 (79.1%) as patients with cardiac arrest. The mean age of these cardiac arrest patients was 68 ± 13 years, with a male predominance (51/68; 75.0%). Return of spontaneous circulation was recorded in 49 patients (72.1%). Over one-third of the cardiac arrest patients (27/68) were alive after 30 days with favourable neurological outcome. The patients who survived the first year lived also to 5 years after the event with favourable neurological and functional status.

**Conclusions:**

The in-hospital cardiac arrest incidence on a large tertiary Swiss university hospital was 1.56 per 1000 patient admissions. After a cardiac arrest, about a third of the patients survived to 5 years with favourable neurological and functional status. Alarms unrelated to life-threatening situations are common and need to be taken into count within a low-threshold alarming system.

*Trial Registration*: The trial was registered in clinicaltrials.gov (NCT02746640).

**Supplementary Information:**

The online version contains supplementary material available at 10.1186/s13049-021-00931-0.

## Introduction

The incidence of in-hospital cardiac arrests (IHCAs) has been reported to be from 0.78 to 4.60 per 1000 patient admissions [1–7]. Despite immediate treatment of IHCAs with high-quality cardiopulmonary resuscitation (CPR) [8, 9], mortality remains high [10]. Patient survival to hospital discharge or up to 30 days after an IHCA has improved over recent years, and has been reported to be around 25% in the United States and up to 35% in European countries. Favourable neurological outcomes were observed in 85% of the survivors in the United States and up to 95% of the survivors in European countries [5, 10, 11]. Long-term survival of these patients has also improved over the past 20 years, although good functional outcomes after 1 year remain at around 13% for IHCA survivors [12].

Fast and competent interventions based on current resuscitation guidelines are essential for increased survival with good neuropsychological outcome after cardiac arrest [13, 14]. As well as local implementation of international resuscitation recommendations [15] and guidelines [13, 14], potential key factors that influence successful in-hospital resuscitation include: (1) a focus on prevention and early recognition of cardiac arrest, with immediate start of basic life support (BLS) [16]; (2) immediate activation of emergency responses to IHCAs, to provide early high-quality advanced life support (ALS) [17]; (3) a high-performing hospital-wide rapid response system consisting of a cardiac arrest team and/or medical emergency team that are organised, well trained, and available 24 h per day, 7 days a week [18–21]; (4) state-of-the-art post-resuscitation care [22]; (5) performance-driven debriefings [23, 24]; and (6) recording of the institution resuscitation success, with reporting of the results. These measures contribute to further optimisation of local procedures and maintain adherence to current guidelines [5, 21].

The Bern University Hospital is one of the largest tertiary acute care hospitals in Switzerland, and it treats about 47,000 inpatients and over 520,000 outpatients annually [25]. It is also a certified referral cardiac arrest centre [26]. Until recently, there was no central documentation of the cardiac arrest team interventions, and no indication of their efficiency within the hospital. Until now, there is no analysis of the annual number of IHCAs, and the resuscitations, outcomes and survival rates of these patients.

Therefore, this prospective single-centre observational study aimed to systematically record and analyse all of the cardiac arrest team interventions in a large Swiss University Hospital over 1 year, focusing specifically on the early phase of the chain of survival. Our findings address the gap in knowledge regarding (1) a large proportion of the Swiss population, (2) the reasons behind the “weakest links” in the chain of survival in this population, and (3) data on functional outcomes and health-related quality of life of cardiac arrest survivors over five years. Our findings can also provide insight into potential challenges in the alarming system, including false alarms and non-technical barriers during resuscitation.

## Methods

### Ethics Commission approval and registration

The study protocol was approved by the Ethics Commission of the Canton of Bern (KEK Nr: 108/15), and was prospectively registered with ClinicalTrials.gov (NCT02746640). The need for informed consent was waived by the Cantonal Ethics Committee. During the follow-up, the purpose of the study was explained to all IHCA survivors or their legal guardian by a member of the study team, and all of them agreed to participate in further voluntary follow-ups per phone without compensation.

### Setting

In case of a cardiac arrest or an acute life-threatening event either in ward patients or hospital visitors at the Bern University Hospital, the cardiac arrest team is activated by the Department of Anaesthesiology and Pain Medicine (for adults) or by the paediatric Intensive Care Unit (ICU) (for children under age 16). Adult ward patients with a non-life-threatening but rapidly deteriorating clinical conditions are primarily referred to the medical emergency team (MET), activated from the adult ICU. All three teams are available 24 h per day, 7 days a week.

The cardiac arrest team for adults is composed of a board-certified anaesthesiologist and a certified anaesthesia nurse who provide high-quality ALS conforming to the guidelines of the European Resuscitation Council (ERC). Quality is assured through education and frequent training. The cardiac arrest team is summoned via a centralised hospital-wide telephone number (“9999”) or by pushing red “cardiac arrest” buttons located throughout the hospital. If an alarm is triggered via the “cardiac arrest” button, there is no communication between the cardiac arrest team and the alarming party. Once activated, the cardiac arrest team carries a resuscitation cart with all equipment necessary for ALS monitoring and treatment at the scene. All of the hospital staff are trained to deliver high-quality BLS according to the ERC guidelines and are instructed in the use of an automated external defibrillator (AED). AEDs are placed at various locations within the hospital campus, and can be used until the cardiac arrest team takes over.

Cardiac arrests that occur in adult and paediatric ICUs, the Cardiac Catheterisation Laboratory, Emergency Rooms or Operating Rooms are treated primarily by the professionals working in these settings. However, the cardiac arrest team can be summoned to these areas as well.

### Participants

We included all adult IHCA team interventions from 1 March, 2015, to 28 February, 2016. We excluded paediatric patients (< 16 years), patients with out-of-hospital cardiac arrests admitted to the Emergency Room under ongoing CPR, and cardiac arrests in the paediatric and adult ICUs.

On hospital admission, the “Do Not Attempt Resuscitation” (DNAR) order is individually discussed between every patient (or their legal guardian) and the treating physicians. For our study, all participants previously expressed their preference regarding DNAR.

### Procedures and measures

During the day, the data were collected by an observer who accompanied the cardiac arrest team, not involved in the treatment. This member of the research team prospectively recorded all of the research-related data on an Utstein-style case report form [27], adapted for IHCAs (Additional file 1: Digital Supplemental Content 1). The observer could report more than one reason per case for an alarm. For IHCAs during the night, the case report form was filled in by the cardiac arrest team, and the medical team leader was interviewed about the event the following day. The observer recorded ‘Return Of Spontaneous Circulation’ (ROSC) during resuscitation when it occurred for at least 1 min, and ‘sustained ROSC’ was defined as no further chest compressions needed for at least 20 min [28]. All patients with IHCA treated by the cardiac arrest team were assessed neurologically with the Glasgow Outcome Scale (GOS) [29, 30], either immediately after resuscitation on scene or on arrival in the ICU, and again 24 h after the cardiac arrest. Target Temperature Management for post-IHCA treatment in the ICU was performed with the normothermia protocol, with early treatment of fever (defined as a body temperature ≥ 37.8 °C). In the ICU, Target Temperature Management and sedation were administered to every patient, according to local guidelines. Sedation interruption was started 24 h after IHCA, to allow for a daily neurological assessment.

At 30 days and 1 year from the cardiac arrest, patients were contacted by telephone for a 30-min interview to assess their neurologic status, using the GOS, and their functional status, using the Short-form-12 Health Survey (SF-12) [31, 32]. After 5 years, they were again contacted by telephone and the GOS was reassessed. All of the patient data were recorded separate from the hospital information system, and stored coded in a password-protected departmental electronic research database, according to the Swiss Federal Human Research Act.

### Instruments

The GOS was used to categorise the neurological outcomes. In brief, the GOS has five categories, where the higher values define better neurological outcomes, as: ‘Death’ (score 1), ‘persistent vegetative state’ (score 2), ‘severe disability’ (score 3), ‘moderate disability’ (score 4) and ‘low disability’ (score 5). For the purpose of this study, GOS 2 and 3 were considered as ‘poor neurological outcome’, while GOS 4 and 5 were considered as ‘favourable neurological outcome’. Sedated patients during post-resuscitation care were not assessed with GOS.

Health-related quality of life was assessed with the SF-12, which comprises 12 questions that define two core dimensions, as the ‘Mental component summary’, and the ‘Physical component summary’, with each calculated on a scale of 0 to 100. These scores are age dependent, and they describe better health-related quality of life as the values increase [28, 31].

### Statistical analysis

Statistical analysis was performed using Stata version 14 (StrataCorp, Texas, USA) and SPSS 27 (IBM Corp., New York, USA). Categorical variables were described as absolute numbers, and relative frequencies as percentages. Continuous variables were described as means ± standard deviation (SD), or median and interquartile range (IQR) for non-normally distributed data. Student’s t-tests were used to compare continuous parametric data, and Mann–Whitney or Kruskal–Wallis tests for non-parametric data. Categorical variables were compared with chi-squared tests or Fisher’s exact tests. The significance level of probability was defined as ≤ 0.05.

## Results

**Fig. 1:**
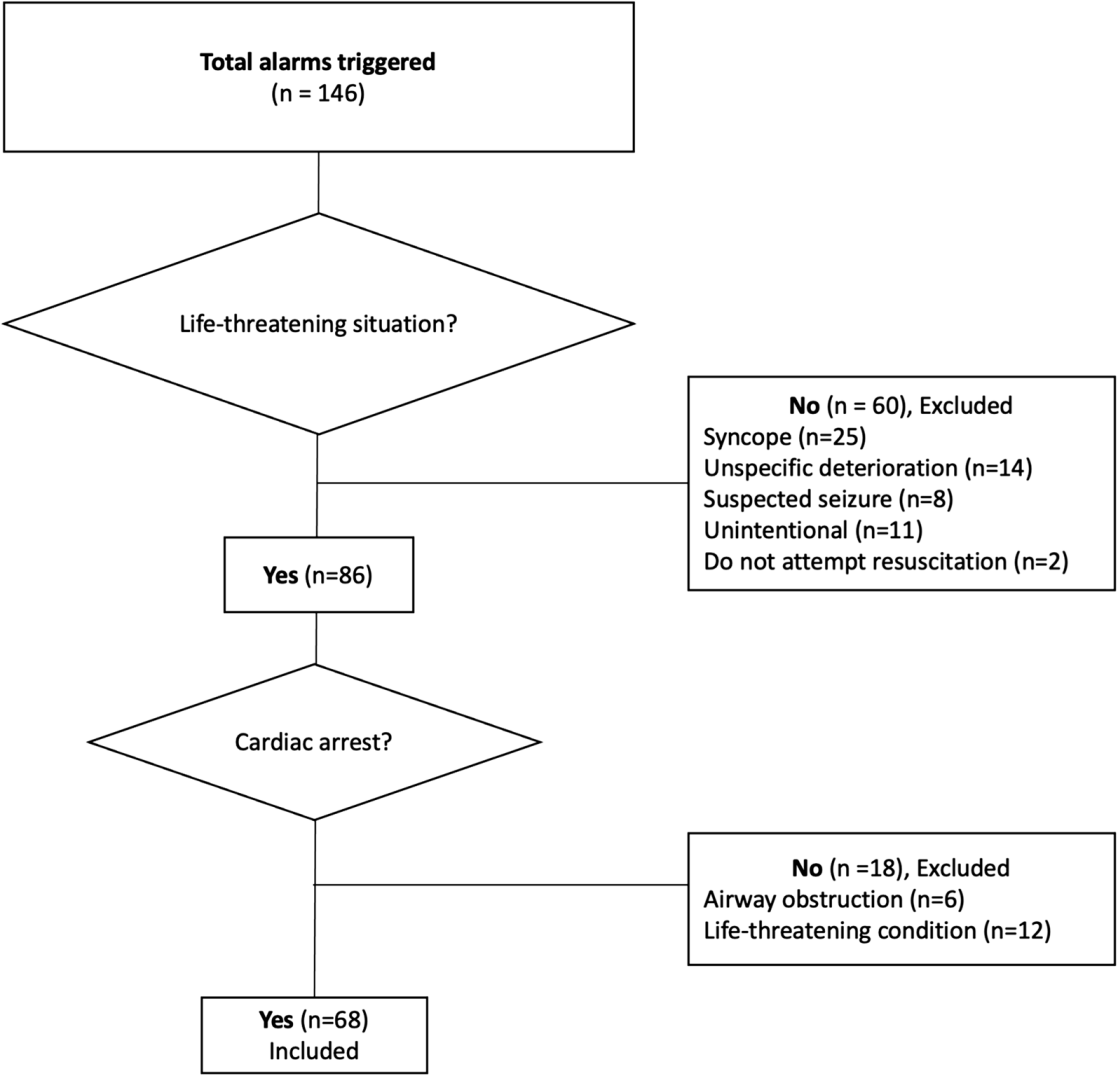
Study flowchart

In all, 146 cardiac arrest team alarms were recorded for the 1-year study period (Table 1, Fig. 1). Of these, 86 (58.9%) were considered acute life-threatening alarms. For 23 patients (15.8%), the alarms were triggered before they went into cardiac arrest. A total of 100 reasons for the alarms were recorded on the Utstein-style case-report forms (Table 1).

**Table 1:** Indications, locations and reasons for the 146 cardiac arrest team alarms

Indication/ location/ reason	Patients [n (%)]
With life threatening conditions	86 (58.9)
Cardiac arrest	68 (46.6)
Acute airway problem	6 (4.1)
Other life-threatening conditions	12 (8.2)
With non-life-threatening conditions	60 (41.1)
Syncope	25 (17.1)
Unspecific deterioration of clinical status	14 (9.6)
Suspected seizure	8 (5.5)
Do not attempt resuscitation order	2 (1.4)
Unintentional activation	11 (7.5)
Locations	
Central campus building	107 (73.3)
Wards	66 (45.2)
Cardiac Catheterisation Laboratory	28 (19.2)
Emergency Room	11 (7.5)
Operating Room	2 (1.4)
Peripheral campus pavilions	31 (21.2)
Not documented	8 (5.5)
Reason for cardiac arrest team alarms^a^	100 (100)
Ongoing cardiopulmonary resuscitation	49 (49.0)
Heart rate < 40 bpm or > 140 bpm	16 (16.0)
Glasgow Coma Scale decrease ≥ 2 points	8 (8.0)
Blood pressure < 90 mmHg or rise from baseline > 40 mmHg	8 (8.0)
Respiration rate < 6 bpm or > 35 bpm	8 (8.0)
Peripheral oxygen saturation (SpO_2_) < 90%	4 (4.0)
Seizure	1 (1.0)
Seriously worried about patient^‡^	3 (3.0)

^a^Cases can accumulate multiple reasons

^‡^Only if no objective reason could be defined

Sixty of the resuscitation alarms (41.1%) were not related to life-threatening conditions. The main medical diagnosis of these were 25 (17.1%) patients suffering from syncope and 14 (9.6%) with unspecific deterioration of clinical status. Eleven alarms (7.5%) were triggered unintentionally (by children, facility personnel, or during construction work). Ten alarms (6.9%) were considered as miscommunication, as the MET should have been called instead of the cardiac arrest team.

Most of the alarms came from the central campus building (n = 107; 73.3%), while 31 (21.2%) came from peripheral campus pavilions. The locations of eight alarms (5.5%) were not recorded. In the central campus building, the alarms came from wards (n = 66; 45.2%), the Cardiac Catheterisation Laboratory (n = 28; 19.2%), the Emergency Room (n = 11; 7.5%), and the Operating Room (n = 2; 1.4%).

Of the 60 alarms that were not related to life-threatening situations, significantly more came from peripheral campus pavilions (n = 20/31; 64.5%) compared to the central campus building (n = 40/107; 37.4%; *p* = 0.002). Overall, for all of the alarms, the mean time between an alarm and the arrival of the cardiac arrest team was 3.0 ± 1.6 min.

### In-hospital cardiac arrests

With 68 IHCAs recorded, this corresponded to an incidence of 1.56 in 1000 admissions (admissions during the study year: 43,697). The descriptive characteristics of the patients who experienced these 68 IHCAs are summarised in Table 2.

**Table 2:** Demographic features and characteristics of the 68 patients with in-hospital cardiac arrests

Demographic	Location		*p*
	Wards	Cardiac catheterisation laboratory/emergency room/operating room	
Total patients(n)	40	28	
Male [n (%)]	25 (62.5)	24 (85.7)	NS
Mean age (years)	63.0 ± 15.8	71.9 ± 12.3	**0.014**
Arrest witnessed [n (%)]	26 (65.0)	28 (100)	
Time to cardiac arrest team arrival (min)	3.4 ± 2.0	2.2 ± 0.8	**0.005**
Initial rhythm [n (%)]			
Shockable	24 (60.0)	19 (67.9)	NS
Non-shockable	6 (15.0)	8 (28.6)	NS
Reason for cardiac arrest [n (%)]			
Cardiac	21 (52.5)	23 (82.1)	**0.012**
Pulmonary	5 (12.5)	1 (3.6)	NS
Neurological/stroke	1 (2.5)	0	NS
Bleeding	2 (5.0)	2 (7.1)	NS
Unknown	11 (27.5)	2 (7.1)	NS
STEMI diagnosed [n (%)]	2 (5.0)	8 (28.6)	NS
Time to ROSC (min)	7.2 ± 8.4	9.6 ± 7.0	NS
Survival [n (%)]			
Immediate	20 (50.0)	23 (82.1)	**0.04**
At 24 h	17 (42.5)	15 (53.6)	NS
At 30 days	12 (30.0)	15 (53.6)	NS
At 1 year	9 (22.5)	13 (46.4)	**0.037**
At 5 years	7 (17.5)	13 (46.4)	**0.015**

Bold values are statistically significant (*p* ≤ 0.05)

STEMI, ST elevation myocardial infarction; ROSC, return of spontaneous circulation

For 55 of these IHCA alarms (80.9%), the cardiac arrest was directly witnessed by a bystander. In 46 of all IHCA alarms (67.6%), chest compressions and bag-mask ventilation were already being performed on arrival of the cardiac arrest team, in 24 of the above 46 patients (52.2%), the self-adhesive pads of an AED had already been attached, and in 13 of these later 24 patients (54.2%) a shock has been delivered by the BLS team prior to the arrival of the cardiac arrest team. For 5 of all IHCAs (7.4%), only chest compressions were being delivered, and in another 5 (7.4%), no CPR had been attempted.

For the 40 (58.8%) ward patients who suffered an IHCA, the cardiac arrest team took 3.4 ± 2.0 min to reach them, which was significantly longer than for the 28 patients (41.2%) who were in the Cardiac Catheterisation Laboratory, Emergency Room or Operating Room (2.2 ± 0.8 min; *p* = 0.005). Comparing these two patient groups further, although those with IHCAs on wards were significantly younger (63.0 ± 15.8 years vs. 71.9 ± 12.3 years; *p* = 0.014), for the patients where the IHCAs occurred in the Cardiac Catheterisation Laboratory, Emergency Room or Operating Room, they had sustained ROSC more frequently (20/40 vs. 23/28; 50.0% vs. 82.1%; *p* = 0.040) and showed greater survival after 1 year (9/40 vs. 13/28; 22.5% vs. 46.4%; *p* = 0.037), although this was not accompanied by better neurologic or functional outcomes.

The patient neurological outcomes for the various recorded periods after resuscitation are summarised in Table 3. Overall, almost three quarters of these patients (n = 49/68; 72.1%) had ROSC during CPR, and 43 patients (63.2%) initially survived (GOS > 1). Eleven patients were sedated (16.2%) after ROSC and therefore could not be assessed at this point. Twenty-three patients (33.8%) treated by the onsite BLS team showed already sustained ROSC with favourable neurological status (moderate to low disability: GOS 4, 4/68 [5.9%]; GOS 5, 19/68 [27.9%]) before arrival of the cardiac arrest team. The remaining immediate surviving patients (n = 20/68; 29.4%) treated by the cardiac arrest team with sustained ROSC showed, on arrival at the ICU, neurological outcomes of persistent vegetative state (GOS 2: n = 3/68 [4.4%]) and severe neurological status (GOS 3: n = 6/68 [8.8%]).

**Table 3:** Outcomes for the 68 patients following their in-hospital cardiac arrests

Outcome	Time from post cardiac arrest				
	Immediate	24 h	30 d	1 y	5 y
ROSC at least 1 min during CPR [n (%)]	49 (72.1)	–	–	–	
Sustained ROSC/ overall survival [n (%)]	43 (63.2)	32 (47.1)	27 (39.7)	22 (32.4)	20 (29.4)
Glasgow Outcome Scale Scores [n (%)]					
1 (dead)	25 (36.8)	11 (16.2)	5 (7.4)	5 (7.4)	0
2–3 (poor outcome)	9 (13.2)	5 (7.4)	2 (2.9)	0	0
4–5 (favourable outcome)	23 (33.8)	24 (35.3)	23 (33.8)	20 (29.4)	20 (29.4)
Not assessable (sedated)	11 (16.2)	3 (4.4)			
Not assessable (language barriers)			2 (2.9)	2 (2.9)	
Short-form-12 Health Survey (mean ± SD)					
Physical Component Summary	–	–	42.8 ± 7.7	47.0 ± 8.6	–
Mental Component Summary	–	–	47.0 ± 13.1	53.4 ± 7.4	–

*ROSC* return of spontaneous circulation, *CPR* cardiopulmonary resuscitation

### Data on follow-up

Twenty-four hours after the IHCAs, nearly half of these 68 patients were still alive (n = 32; 47.1%). Three patients were sedated (9.4%) and therefore could not be assessed at this point. Favourable neurological outcomes (i.e., GOS 4, 5) were recorded for the majority of these patients (n = 24/32; 75.0%), although some had severe disability (GOS 3: n = 4/32; 12.5%), and one patient was in a vegetative state (GOS 2: 3.1%). Eleven patients (16.2%) who showed immediate post-IHCA survival died within the first 24 h.

At 30 days, over one-third of the patients were still alive (n = 27/68; 39.7%). Excluding two patients alive at follow up (at 30 days, 1 year and 5 years) that could not be assessed neurologically due to language barriers (n = 2/68; 2.9%), almost all of the patients who remained alive (n = 23/27; 85.2%) showed favourable neurological status (GOS 4: n = 3/27, 11.1%; GOS 5: n = 20/27, 74.1%), with only two of these 27 (7.4%) in a severe status (GOS 3). Five patients who had survived the first 24 h died within the first 30 days (n = 5/68; 7.4%).

One year after IHCA, there were 20 patients (29.4%) still alive, who also showed favourable neurological outcomes (GOS 4, n = 3, 4%, GOS 5, n = 17, 25%); none of these alive patients were recorded with GOS 2 or 3. Five patients (7.4%) had died from 30 days to the end of the first year from these IHCAs. Again, two patients (2.9%) could not be assessed due to language barriers.

As for the SF-12 assessments of the alive and assessable patients after 30 days (n = 23/27), a comparison with the Physical Component Summary score of a healthy sample of the Swiss population (49.8 ± 8.6) [32] showed a lower mean value (42.8 ± 7.7) (Table 3). However, this difference balanced out at 1 year after their IHCAs (47.0 ± 8.6). For the Mental Component Summary score after 30 days, compared to healthy volunteers (46.3 ± 10.1), no significant difference was seen (47.0 ± 13.1). These patients also showed a small, but not significant, increase in their Mental Component Summary score after 1 year (53.4 ± 7.4).

Five years after IHCA, the 20 assessable one-year survivors were contacted again. All 20 patients (29.4%) were still alive and all demonstrated favourable neurological outcomes (GOS 4, n = 3, 4%, GOS 5, n = 17, 25%). None of these patients had a GOS 2 or 3, and no IHCA survivor had died between first and fifth year after.

## Discussion

This study analysed all adult cardiac arrest team interventions at a large Swiss university hospital over 1 year. The incidence of IHCA was 1.56 per 1,000 admissions. Our data is in agreement with other studies [1–7, 33] as European cohorts show an incidence ranging between 1.5 and 2.8 per 1,000 hospital admissions [5]. Additionally, the 30-day (33.8%) and 1-year survivors (29.4%), and their neurological and functional outcomes, were a little higher than reported in other studies [2–7, 33, 34]. Published survival rates at 30 days / hospital discharge vary from 15 to 34% [5]. There are several reasons that could explain our high survival rate. First, the majority of our cohort had a shockable first rhythm. Not surprisingly, we had greater attainment of ROSC and increased survival in patients presenting with a shockable rhythm during cardiac arrest, and this subset of patients included many of those arresting in the Cardiac Catheterisation Laboratory, Emergency Room or Operating Room. Previous studies reported IHCA distribution of shockable rhythms around 20% compared to non-shockable rhythms around 80%. Shockable rhythms are associated with better outcomes (up to 40%) compared to non-shockable rhythms (around 12%) [10, 35, 36]. A main reason for the high prevalence of shockable rhythms in our cohort could be the inclusion of patients who are highly monitored, especially in the cardiac catheterization laboratory, the male predominance through our cohort [35], and finally the low numbers of IHCA.

A 2018 systematic review that analysed more than one million IHCAs from 1992 to 2016 reported an overall pooled 1-year survival of 13%, with a range of 6% to 28%, and large between-study variability [12]. Although our findings are in agreement with such reports, the available literature is often from single centres, making generalizability difficult, and ultimately all patients who die in hospital die from a cardiac arrest [5]. Additionally, several factors have been associated with survival, including the initial rhythm, the place of arrest and the degree of monitoring at the time of collapse, which makes studies challenging to compare.

Finally, our relatively high survival rate may be explained in part by the DNAR-order procedure at the Bern University Hospital and the presence of a well-functioning MET team, which transfers deteriorating ward-patients to higher levels of care. This is corroborated by studies showing that in countries where withdrawal of life sustaining treatment is common, a favourable neurological outcome is seen in over 90% of IHCA patients [5].

### False alarms

Perhaps surprisingly, 41.1% of all of this study’s cardiac arrest team alarms were unrelated to life-threatening events, with only about half of all of the alarms actually activated for a cardiac arrest. Life-threatening situations like cardiac arrest or respiratory failure due to acute airway obstruction need immediate and competent help for better survival. Therefore, the use of a low-threshold alarm system [19, 37] that can be activated by any healthcare worker in a hospital, or even by a visitor to the hospital, allows for rapid rescue interventions. The downside of such a low-threshold alarm system is that alarms unrelated to life-threatening events (i.e., ‘false alarms’) can also be triggered. Dukes et al. [19] underlined that such a low-threshold alarm system is important for top performing hospitals in terms of IHCAs.

This study also revealed syncope as the main medical reason for alarms unrelated to life-threatening situations. From a bystander's perspective the difference between a syncope and a collapse due to cardiac arrest can be initially difficult to discriminate. For the Bern University Hospital, hospital staff are advised and encouraged to activate the cardiac arrest team as soon as possible, aiming to shorten the time to arrival and therefore improve the patient`s outcome [5]. Our findings are in line with other reporting on false cardiac arrest alarms [38], where the most frequent reasons for false alarms were collapse or vasovagal syncope. Surprisingly, patients with a false alarm had lower survival rates than the general hospitalised population. These findings help to raise awareness that even alarms that are not life-threatening should be taken seriously, as these patients may have higher mortality. It is better to alert the cardiac arrest team when they’re not needed, than to fail to notify them when they are. The burden to delay the activation of the cardiac arrest team should be low as the potential harm of inappropriate activation is negligible. The low-threshold alarm system in this large Swiss university hospital might explain the high proportion of purported cardiac arrest alarms that were unrelated to life-threatening events.

On the other hand, unintentional alarming through “cardiac arrest” buttons is rather difficult to avoid, even if these buttons are covered with a plastic barrier to lift before activation. To fulfil their purpose, these buttons need to be easily reachable in locations where telephones are not available. This can also make them reachable for children. Finally, visitors often do not know their purpose and could misinterpret them as an option to call the responsible nurse of the ward.

Finally, miscommunication was identified as a common reason for alarming the cardiac arrest team instead of the MET. As the Bern University Hospital frequently trains and employs new staff, this high personnel turnover with a lack of a proper educational program may be responsible for the unfamiliarity of the activation criteria for the cardiac arrest team or the MET. On the other hand, trained staff is known to communicate suboptimal in emergency situations [39], particularly if their non-technical competencies are not refreshed [40].

### Role of teaching and “boost refreshers”

We verified that the cardiac arrest team took more time to reach patients in peripheral wards, although this did not influence patient survival. For hospitals that cover large areas of land or are spread over several floors, reaching patients can pose a problem. One way to overcome this is the constant teaching and training of the resuscitation competence of ward personnel, with the aim being to provide early high-quality BLS. In most cases, the ward staff correctly applied the BLS algorithm, which resulted in sustained ROSC by the time the cardiac arrest team arrived for about one-third of these patients. Some reports of incomplete (n = 5, 7.4%) or absent (n = 5, 7.4%) BLS prior to arrival of the cardiac arrest team are of concern. Our findings imply that hospitals should promote continuous efforts to educate the ward staff, focusing specifically on overcoming the barriers to performing BLS. Lauridsen et al. categorized barriers and facilitators for in-hospital resuscitation in four different domains (treatment, teamwork, leadership, and communication) [39]. In our study we can confirm some of the findings regarding barriers: for “treatment”, occasionally BLS was performed without appropriate equipment (missing face-mask ventilation or AED) and there were cases of missing handover information (especially regarding comorbidities of the patient). For “BLS teamwork”, overcrowded scenes (especially during day-shifts on wards) were reported, and role allocation was often not clear. For “leadership”, the incoming cardiac arrest team was often uncertain who was leading the BLS team. Finally, “communication within the BLS team” was found to be rather loud, unclear and not in closed loops.

We also confirm some facilitators. The cardiac arrest team members are familiar with the resuscitation cart, and they are trained to fill more than one role in a resuscitation team with the ability to change roles performing ALS. Leadership was clearly defined and they communicated in a closed loop manner.

BLS skills decrease over time from 3 to 12 months after training, and therefore brief and frequent re-training is recommended [41]. While the best timing for re-training is still under debate, a recent study showed that monthly training of CPR skills is highly effective to improve performance [42], although implementation would difficult due to time constraints. Therefore, mandatory yearly short competence refresher courses might be an easy way to ensure delivery of early high-quality BLS [23, 41].

As for telephone alarm systems, the contact person might also be instructed to give advice to the person calling, on how best to deliver BLS until the cardiac arrest team arrives. In OHCA setting, CPR instructions delivered by telecommunication dispatchers have been shown to be independently associated with improved survival and improved functional outcome [43], and these are highly recommended by international resuscitation guidelines [13–15]. This might also be a suitable option to ensure correct and effective IHCA treatment. Specific and adapted education will be needed for these in-hospital telephone dispatchers to be able to confirm a cardiac arrest situation, to provide instructions for BLS via the telephone, and to encourage the first rescuer to consider early use of an AED while the cardiac arrest team is on its way. The potential influence of dispatchers who deliver CPR instructions via the telephone should be looked at in the context of IHCA survival, and favourable neurological long-term outcomes should be investigated in future studies.

### Limitations

The major study limitations here were the overall low reported numbers of cardiac arrest team alarms over the 1-year observation period and the loss of some patients during follow-up, mostly because further assessment was not possible due to language barriers. Some cases had missing values in the Utstein-style case report form. This was more frequent for cardiac arrest alarms during late-shifts or night-shifts, where personnel capacity is reduced compared to day-shifts and the cardiac arrest team had to be interviewed retrospectively. This, and the relatively low numbers of ICHA patients, might have influenced our results. Therefore, we avoided any data adjustment, and further interpretation needs to be performed cautiously. Moreover, as IHCAs from paediatric and adult ICUs were not included in the study, we cannot report outcome data for these patients. All in all, these limitations might have resulted in an underestimation of the overall incidence of IHCA at Bern University Hospital. Additionally, because cardiac arrest team members were being observed by an external person, we cannot exclude the possibility of the Hawthorne effect influencing reported team performance. This underlines the importance of reporting and integrating even small amounts of local data into international registries and databases, with the aim of further exploring the incidence, survival and long-term outcomes of IHCAs, and defining national and regional differences.

### Conclusions

This 5-year prospective observational study reports survival data of in-hospital cardiac arrests recorded over one year in one of the largest Swiss tertiary centres and university hospitals. We reported an incidence of IHCAs of 1.56 in 1,000 hospital admissions. The 30-day survival rate was 40%, with 34% having good neurological outcomes. One year later, 32% of IHCA patients remained alive, with 29% having a favourable neurological outcome. Five years after IHCA all assessable survivors (29%) remained with a favourable neurological outcome. To improve patient outcome further, enhanced annual resuscitation competence refresher courses are needed, particularly in large campus areas where cardiac arrest teams need more time to reach patients. High numbers of alarms for patients with non-life-threatening conditions also need to be taken into count within a low-threshold alarm system.

## Supplementary Information

**Additional file 1**. Utstein-style case report form.

## Data Availability

The datasets analysed during the current study are available from the corresponding author on reasonable request, with Ethics Committee approval.

## Abbreviations

AED: Automated external defibrillator
ALS: Advanced life support
BLS: Basic life support
CPR: Cardiopulmonary resuscitation
ERC: European Resuscitation Council
GOS: Glasgow Outcome Scale
ICU: Intensive care unit
IHCA: In-hospital cardiac arrest
ROSC: Return of spontaneous circulation
SF-12: Short-form-12 Health Survey
