# Supplementary material for: Survival and long-term outcomes following in-hospital cardiac arrest in a Swiss university hospital: a prospective observational study
Source: Scand J Trauma Resusc Emerg Med. 2021 Aug 11;29:115. doi: 10.1186/s13049-021-00931-0 (PMC8359113; doi:10.1186/s13049-021-00931-0)
Supplement: Supplementary file 1 — Additional file 1. Utstein-style case report form. [file 13049_2021_931_MOESM1_ESM.pdf]

CPR-Protocol

Time: Alarm until →

Arrival of ALS at Patient:	Chest Compression:	Defibrillation:	Drugs:

Patient data

Participant-ID:

Gender:	◯Male ◯Female
Day:	
Time:	
Type of survey:	◯Witnessed ◯Enquiry
Location at Inselspital/Bern University Hospital:	
Initial reason for hospital attendance:	

Comments:

Alarm

Dispatcher identified presence of cardiac arrest	◯Yes ◯No
Dispatcher provided CPR instructions	◯Yes ◯No
Witnessed cardiac arrest	◯Bystander wit. ◯BLS wit. ◯Not wit.
Bystander or BLS Response	◯Compression ◯Ventilation ◯No CPR<br>◯AED/Defi. used → ◯Shock delivered
Reason for alarm	◯Respiratory Rate < 6 or > 35/min ◯O2 sat. < 90

◯Blood pressure < 90 or increase > 40 mmHg ◯Heart rate < 40 or > 140/min
◯GCS decrease ≥ 2 ◯Epileptic seizure ◯Seriously worried about patient

False Alarm? (no CPR) ◯Yes ◯No

If Yes → please explain:

B

Ventilation:	◯Yes ◯No
Airway control (please note sequence)	◯Bag-valve-mask ◯Naso-/Oropharyngeal ◯Tracheal tube ◯Supraglottic ◯Surgical ◯None ◯Unknown

C

Cardiac arrest confirmed by ALS team?	◯On arrival ◯Occurred after arrival ◯No → please explain
Monitored rhythm (in order of occurrence)	◯Asystole ◯PEA ◯Pulsless VT ◯VF ◯Normal/SR or no CPR ◯Unknown
Etiology	◯Cardiac ◯Pulmonal ◯Anaphylactic ◯Drug overdose/Intoxikation ◯Sepsis
	◯Neurological/Stroke ◯Trauma ◯Unknown ◯Other → please explain
Comorbidities	◯No ◯Unknown ◯Yes → please explain
Implanted cardioverter-defibrillator	◯Yes ◯No ◯Unknown
Ventricular Assist Device	◯Yes ◯No ◯Not recorded
Presence of STEMI	◯Yes ◯No ◯Unknown
Type of reperfusion attempted	◯Angiography only ◯PCI ◯Thrombolysis ◯None ◯Unknown
Timing of reperfusion attempted	◯Intra-arrest ◯Within 24 h of ROSC ◯> 24 h but before discharge ◯Unknown

Comments:

Resuscitation by ALS team

Resuscitation attempted	◯Yes ◯No
Chest compression	◯Yes ◯No
Mechanical CPR	◯No ◯Yes → Please note type of device:
Defibrillation	◯Yes ◯No
Number of shocks	
External or temporary pacemaker	◯Yes ◯No
Drugs given	◯Adrenaline ◯Amiodarone ◯Atropine ◯Vasopressin (ADH) ◯Noradrenaline ◯Other:
Primary vascular access used during arrest	◯PVC ◯CVC ◯IO ◯IM ◯Endotracheal ◯Unknown ◯Other:
CPR quality measured	◯No ◯Yes → please explain/note type of device:
ROSC at any time achieved	◯Yes ◯No ◯Unknown
Targeted oxygenation/ventilation after ROSC	◯Yes ◯No ◯Unknown
Targeted blood pressure management after ROSC	◯Yes, target: mmHg ◯No ◯Unknown
Targeted temperature management	◯Yes, target: °C ◯No ◯Unknown
pH	◯No ◯Yes → please note value
Lactate	◯No ◯Yes → please note value
Glucose	◯No ◯Yes → please note value
Extracorporeal life support (ECLS)	◯Intra-arrest/Before ROSC ◯After ROSC ◯Not used ◯Unknown
Intra-aortic balloon pump (IABP)	◯Yes ◯No ◯Unknown
12-Lead ECG after ROSC?	◯Yes ◯No ◯Unknown

After Resuscitation

Survived event (until arriving at ICU) (GOS)	◯1. Death ◯2. Persistent vegetative state ◯3. Severe disability ◯4. Moderate dis. ◯5. Low dis.
24 h survival (GOS)	◯1. Death ◯2. Persistent vegetative state ◯3. Severe disability ◯4. Moderate dis. ◯5. Low dis.
30 days survival or survival to discharge (GOS)	◯1. Death ◯2. Persistent vegetative state ◯3. Severe dis. ◯4. Moderate dis. ◯5. Low dis.
Quality of life after 30 days or at discharge (SF12)	PCS: MCS:
12 months survival (GOS)	◯1. Death ◯2. Persistent vegetative state ◯3. Severe disability ◯4. Moderate dis. ◯5. Low dis.
Quality of life after 12 month (SF12)	PCS: MCS:
Cause of death (if known):	

GOS: Glascow Outcome Scale SF12: Short Form 12 Health Survey PCS: Physical Component Summary MCS: Mental Component Summary
